# Diagnostic value of urodynamic bladder outlet obstruction to select patients for transurethral surgery of the prostate: Systematic review and meta-analysis

**DOI:** 10.1371/journal.pone.0172590

**Published:** 2017-02-27

**Authors:** Myong Kim, Chang Wook Jeong, Seung-June Oh

**Affiliations:** 1 Department of Urology, University of Ulsan College of Medicine, Asan Medical Center, Seoul, Republic of Korea; 2 Department of Urology, Seoul National University College of Medicine, Seoul National University Hospital, Seoul, Republic of Korea; University of Oklahoma Health Sciences Center, UNITED STATES

## Abstract

**Purpose:**

To investigate the diagnostic value of urodynamic bladder outlet obstruction (BOO) in the selection of patients for transurethral surgery of the prostate.

**Materials and methods:**

We systematically searched online PubMed, Embase, and Cochrane Library databases from January 1989 to June 2014.

**Results:**

A total of 19 articles met the eligibility criteria for this systematic review. The eligible studies included a total of 2321 patients with a median number of 92 patients per study (range: 12–437). Of the 19 studies, 15 conducted conventional transurethral prostatectomy (TURP), and 7 used other or multiple modalities. In urodynamic bladder outlet obstruction (BOO) positive patients, the pooled mean difference (MD) was significant for better improvement of the International Prostate Symptom Score (IPSS) (pooled MD, 3.48; 95% confidence interval [CI], 1.72–5.24; p < 0.01; studies, 16; participants, 1726), quality of life score (QoL) (pooled MD, 0.56; 95% CI, 0.14–1.02; p = 0.010; studies, 9; participants, 1052), maximal flow rate (Q_max_) (pooled MD, 3.86; 95% CI, 2.17–5.54; p < 0.01; studies, 17; participants, 1852), and post-void residual volume (PVR) (pooled MD, 32.46; 95% CI, 23.34–41.58; p < 0.01; studies, 10; participants, 1219) compared with that in non-BOO patients. Some comparisons showed between-study heterogeneity despite the strict selection criteria of the included studies. However, there was no clear evidence of publication bias in this meta-analysis.

**Conclusions:**

Our meta-analysis results showed a significant association between urodynamic BOO and better improvements in all treatment outcome parameters. Preoperative UDS may add insight into postoperative outcomes after surgical treatment of benign prostatic hyperplasia.

## Introduction

Traditionally, the primary goal of treatment of benign prostatic hyperplasia (BPH) has been to lessen the bothersome lower urinary tract symptoms (LUTS) caused by prostatic enlargement[1,2]. Surgery is the most invasive option for BPH treatment which can cause irreversible complications[3]. To ensure a better outcome, proper indicators for surgical intervention should be selected. The most recent international treatment guidelines commonly recommend that a surgical intervention should be considered in BPH patients with failure to treatment with oral medications or with complicated LUTS[1,2].

The mechanism for surgery is based on the classic bladder outlet obstruction (BOO) model. Enlarged prostate tissue causes obstruction and increases the urethral resistance to flow, and therefore requires higher intravesical pressure to void[2]. The urodynamic study (UDS) is the only gold standard for the diagnosis of BOO[4]; however, invasiveness, cost, and morbidity of UDS limit its clinical use[5]. In this regard, most guidelines recommend UDS for male LUTS evaluation only in specific situations such as, prior to surgery, previous unsuccessful treatment, functional cystometric capacity < 150mL, post-void residual urine (PVR) > 300mL, patient too young (< 50 years) or too old (> 80 years) for surgery[1], or maximal flow rate (Q_max_) > 10mL/s (relative BOO)[2]. However, most of those recommendations are supported by very low level of evidences (LEs) (all LE = 3)[1]. To our knowledge, there have been no randomized studies regarding the usefulness of UDS for guiding clinical application in male LUTS. There are no published randomized controlled trials in men with LUTS that compare the standard investigations such as symptom score or uroflowmetry (UFM) with UDS[1]. Moreover, the utility of performing UDS before transurethral surgery has rarely been studied in a systemic fashion.

Because reports on the diagnostic value of urodynamic BOO for LUTS in men are few, a combination of these data to reach a reasonable conclusion is necessary. The objective of the present study was to conduct a systematic review and meta-analysis of published literature investigating the diagnostic value of urodynamic BOO in the selection of patients for transurethral surgery of the prostate and to provide a higher LE for guiding practical use of UDS in BPH patients.

## Materials and methods

### Search strategy for relevant studies

The whole process for this systematic review and meta-analysis was conducted according to the study protocol approved by all authors and followed the up-dated versions of MOOSE and PRISMA recommendations (S1 and S2 Checklists) [6,7]. We systematically searched online PubMed, Embase, and Cochrane Library database up to June 2014. Our overall search strategies included terms for UDS (urodynamic, cystometry, and pressure flow study), BPH (benign prostatic hyperplasia, benign prostatic obstruction, and male LUTS), and transurethral surgery (transurethral resection, transurethral incision, vaporization, ablation, and enucleation). Detailed queries for search strategy are presented in S1 Table. Manual search of relevant studies were also performed referring to review articles or original research articles on similar subjects.

### Selection criteria of eligible studies for meta-analysis

The inclusion criteria for our systematic review were as follows: (1) original research articles published in English; (2) studies that included patients with BPH alone; (3) studies that included subjects who underwent transurethral surgery for BPH; (4) studies that included cases preoperatively sub-grouped by the urodynamic criteria of BOO; (5) studies in which outcome parameters were objectively described using standard investigation tools such as the International Prostate Symptom Score (IPSS) or UFM parameters; (6) studies that investigated the association between the urodynamic BOO and improvement of treatment outcome; (7) studies with a definite sample size; and (8) studies that had standard deviation (SD), confidence interval (CI), or other distributional information of outcome parameters. When duplication of patient data was suspected, the most recently published or most informative single article was selected. If the population of the study underwent two or more surgical procedures[8,9], data were processed separately according to the type of surgery. Due to the unavailability of randomized studies, all non-randomized and retrospective studies were included in the systematic review and meta-analysis. Exclusion criteria were as follows: (1) the study could not satisfy the aforementioned inclusion criteria; (2) review articles or letters; (3) laboratory studies such as studies on *ex-vivo* and animal models; and (4) studies with data that were insufficient to estimate the mean and SD of improved outcomes.

To minimize the bias, abstract screening and full text assessment for eligibility were independently performed by all three authors (MK, CWJ, and SJO). All screened abstracts were classified into three categories: (1) not-eligible, (2) unclear, and (3) potentially-eligible. The full texts of “potentially-eligible” and “unclear” studies were obtained and assessed for eligibility. All disagreements among three reviewers were resolved by a consensus meeting.

### Data extraction and quality assessments

The extracted data elements were as follows: (1) overall characteristics of eligible studies, which included the name of first author, publication year, country, recruitment period, study design, population size, type of surgical intervention, urodynamic standards and cut-off value to diagnosis BOO, and quality score for each study; (2) patient characteristics, which included analyzed-population size, mean or median age, time of treatment outcome evaluation, and compared-outcome parameters; and (3) mean improvement of IPSS (ΔIPSS), IPSS-quality of life score (QoL) (ΔIPSS-QoL), Q_max_ (ΔQ_max_), and PVR (ΔPVR) of each subgroup with their SD, after the surgical interventions. Study quality was assessed independently by all three authors using the MINORS (score range: 0–24)[10]. Any disagreement was resolved by discussion.

### Statistical analysis

#### Primary analysis

The improvements of the main outcome parameters (ΔIPSS, ΔIPSS-QoL, ΔQ_max_, and ΔPVR) were compared between the preoperative urodynamic BOO positive and negative subgroups. Owing to the continuous parametric feature of outcomes, the pooled mean difference (MD) was used to summarize each outcome parameters across the eligible studies. A random-effect model was used to obtain the pooled MDs and their 95% CIs, because we hypothesized that the selected eligible studies contains inter-study heterogeneities (*e*.*g*., surgical modalities, period of postoperative outcome evaluation, and urodynamic cut-off values) as well as within-study heterogeneities (*e*.*g*., sampling variation). Mean value and SD of outcome parameters were needed for data integration. For each study, these values were estimated based on the data provided in the publications using previously suggested techniques[11,12]. An observed pooled MD > 0 indicated better improvement of BOO positive group compared to BOO negative, and would be considered statistically significant if the 95% CI did not overlap the pooled MD value of zero, with *p* < 0.05.

#### Subgroup (sensitivity) analysis

Subsequently, we assessed subgroup analysis in patients who underwent conventional transurethral resection of the prostate (TURP) to evaluate the effect of type of surgery performed. Furthermore, subgroup analyses were also performed according to the two dominant criteria for BOO diagnosis (BOO index [BOOI] > 40[13], and linear passive urethral resistance relation [lin PURR] grade ≥ 2, 3, or 4[14]) to evaluate the effects of diagnostic criteria.

#### Assessment of heterogeneity

Heterogeneity was assessed using the heterogeneity х^2^ test (Cochran’s Q-test), a *p* value of > 0.05 indicated the absence of significant heterogeneity[15]. The I^2^ statistic (Higgin H-test) was performed to visualize degree of heterogeneity[16].

#### Publication bias

Possibilities of publication bias were assessed using Funnel plots (Harbord test)[17].

#### Utilized tools

Review Manager (RevMan) version 5.3 (The Nordic Cochrane Center, The Cochrane Collaboration, Copenhagen, Denmark) was utilized for the meta-analysis.

## Results

### Outcome of the selection process

**Fig 1:**
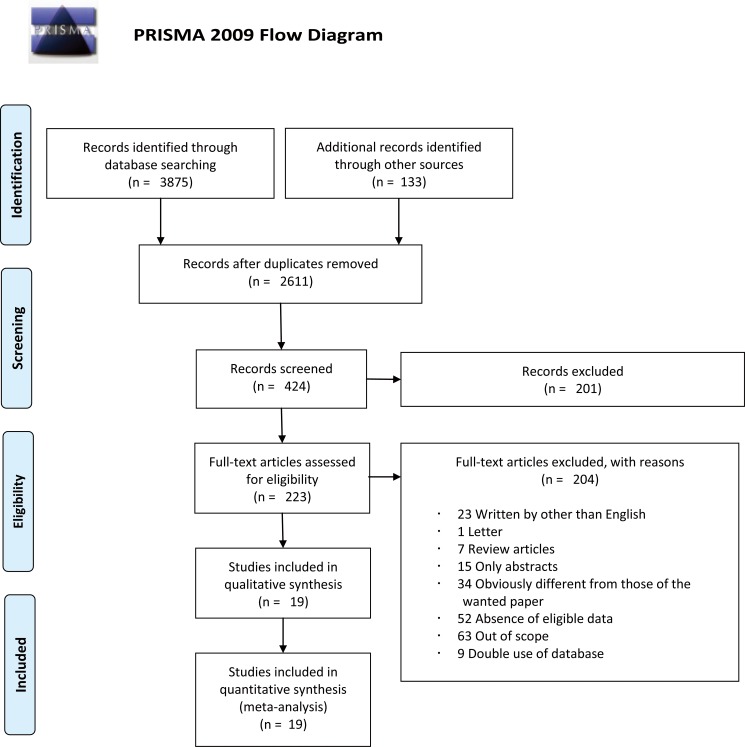
Methodological flow chart of the systematic review.

A flow chart of the whole study selection process is shown in Fig 1. Our search strategy identified 3875 articles (PubMed, 1445 articles; Embase, 2137 articles; Cochrane Library database, 293 articles). Additionally, 133 articles were found by manual searching. After duplicates were removed, 2611 abstracts were independently screened by three authors. After abstract screening, 223 articles were entered to full text assessment for eligibility. After careful review of full articles, 204 were excluded for the following reasons: 23 studies were written in languages other than English, seven were review articles, one was a letter to editor, the full text of the manuscript could not obtained in 15 articles, 63 were out of scope, 34 covered related subjects but could not satisfy the inclusion criteria regarding methodology, 52 lacked eligible data, and nine studies were excluded due to the duplication of the study population. Eventually 19 studies were selected as eligible for the data synthesis[8,9,18–39].

### Characteristics of included studies

Tables 1 and 2 show the general characteristics of eligible studies. The 19 eligible studies included a total of 2321 patients, with a median number of 92 patients per study (range: 12–487). None of the selected studies was a randomized prospective study. Seven of the 19 included studies were non-randomized prospective studies, and the remaining studies had a retrospective design. Patients received TURP in 15 of 19 studies [18,19,24–36], transurethral microwave thermotherapy (TUMT) in one study[20], interstitial laser coagulation (ILC) in one study[23], and multiple intervention modalities in two studies[8,9]. The definition of urodynamic BOO varied among studies. The urodynamic BOO was defined as BOOI > 40 cmH_2_O in nine studies[25,27,28,31,33,34,36,38,39], Lin PURR grade ≥ 4 in four studies[8,20,23,30], Lin PURR grade ≥ 3 in four studies[24,26,29,35], Lin PURR grade ≥ 2 in one study[32], and other definitions in four studies[9,18,19,37]. The median quality score measured by MINORS recorded as 16 (range: 14–18). There was no significant correlation between population size and quality scores (p = 0.231, by Spearman’s correlation analysis) (Table 1). Median or mean ages of study populations were as shown in Table 2. Time of treatment outcome evaluation varied (range: 1.5–144 months). Mean and SD values of ΔIPSS, ΔIPSS-QoL, ΔQmax, and ΔPVR could not be obtained in all studies (Table 2). If the mean and SD values could not be obtained, these values were estimated using other representative and distributional values. Details of the process applied are shown in the S2 Table.

**Table 1:** Main characteristics of eligible studies.

Study	Year	Country	Recruitment period	Study design	Total study population	Type of surgery	Standards of BOO	Cut*-*off	Quality Assessment (0–24)*
Schäfer[18]	1989	Germany	NA	Non-randomized prospective	47	TURP	P_muo_	> 40 cmH_2_O	16
Gormley[19]	1993	Canada	NA	Non-randomized prospective	12	TURP	URA	> 29 cmH_2_O	17
De la Rosette[20]	1996	Multination	1993 –NA	Non-randomized prospective	120	TUMT	Lin PURR	≥ grade 4	18
Ignjatovic[35]	1997	Yugoslavia	NA	retrospective	48	TURP	Lin PURR	≥ grade 3	16
Witjes[8]	1997	Multination	1992 –NA	Non-randomized prospective	487	TURP/PVP/ TUMT	Lin PURR	≥ grade 4	18
Javlé[36]	1998	England	NA	Non-randomized prospective	55	TURP	BOOI	> 40 cmH_2_O	17
Dӕhlin[23]	1999	Norway	1995–1996	retrospective	49	ILC	Lin PURR	≥ grade 4	16
Gotoh[24]	1999	Japan	NA	retrospective	74	TURP	Lin PURR	≥ grade 3	15
Machino[25]	2002	Japan	1992–1999	retrospective	62	TURP	BOOI	> 40 cmH_2_O	14
Porru[26]	2002	Italy	NA	retrospective	45	TURP	Lin PURR	≥ grade 3	15
Hakenberg[27]	2003	Multination	NA	Non-randomized prospective	95	TURP	BOOI	> 40 cmH_2_O	16
Van Venrooij[28]	2003	Netherlands	1996–2002	Non-randomized prospective	132	TURP	BOOI	> 40 cmH_2_O	17
Seki[29]	2006	Japan	1993–2001	retrospective	190	TURP	Lin PURR	≥ grade 3	14
Tanaka[30]	2006	Japan	1995–1997	retrospective	92	TURP	Lin PURR	≥ grade 4	18
Vesely[9]	2006	Sweden	NA	retrospective	231	TURP/TUMT	DAMPF	> 65	14
Han[31]	2008	Korea	NA	retrospective	71	TURP	BOOI	> 40 cmH_2_O	14
Masumori[32]	2010	Japan	1995–1997	retrospective	92	TURP	Lin PURR	≥ grade 2	14
Oh[33]	2010	Korea	2007–2009	retrospective	134	TURP	BOOI	≥ 40 cmH_2_O	16
Min[34]	2013	Korea	2006–2011	retrospective	285	TURP	BOOI	> 40 cmH_2_O	18

BOO, bladder outlet obstruction; NA, not available; TURP, transurethral prostatectomy; P_muo,_ minimal urethral opening pressure; URA, urethral resistance factor; TUMT, transurethral microwave thermotherapy; Lin PURR, linear passive urethral resistance relation; BOOI, BOO index; ILC, interstitial laser coagulation; DAMPF, Detrusor Mean Lin PURR Factor

*Evaluated using Methodological Index for Non-Randomized Studies (MINORS)[10].

**Table 2:** Patient characteristics.

Study	No. of Analyzed patients	Median age, range (or ±SD) (yr)	Type of surgery	Time of outcome evaluation (months)	Compared outcome parameters			
					Symptom score	QoL score	Q_max_ (mL/sec)	PVR (mL)
Schäfer[18]	39	NA	TURP	NA	NA	NA	available	NA
Gormley[19]	12	80 (mean), 72–90	TURP	1.5	NA	NA	available	NA
De la Rosette[20]	120	67. 0 (mean), 45–89	TUMT	6	IPSS	NA	available	available
Ignjatovic[35]	48	NA	TURP	6	IPSS	NA	NA	NA
Witjes (TURP)[8]	87	68.6 (mean), (±8.1)	TURP	6	Frimodt-Møller score	NA	available	available
Witjes (Laser)[8]	83	64.7 (mean), (±7.0)	PVP	6	IPSS	NA	available	available
Witjes (TUMT)[8]	136	66.7 (mean), (±8.3)	TUMT	6	IPSS	NA	available	available
Javlé[36]	53 (BOO)/ 50 (DUA)	68.5 (mean), 55–85	TURP	3	IPSS	NA	available	available
Dӕhlin[23]	24	49, 52–80	ILC	6	IPSS	NA	available	NA
Gotoh[24]	74	73 (mean), 50–86	TURP	1.5–2	NA	NA	available	available
Machino[25]	62	70.3(mean), (±5.4)	TURP	3	IPSS	IPSS-QoL	available	available
Porru[26]	45	66.8 (mean), 52–81	TURP	3–6	IPSS	IPSS-QoL	available	available
Hakenberg[27]	76	74.29 (mean), 46–88	TURP	3	IPSS	NA	available	NA
Van Venrooij[28]	93	65.5 (mean), (±4.1)	TURP	6	IPSS	IPSS-QoL	available	NA
Seki[29]	190	71.3 (mean), (±7.1)	TURP	3, 12	IPSS	IPSS-QoL	available	NA
Tanaka[30]	92	69.7 (mean), 54–87	TURP	3	IPSS	IPSS-QoL	available	available
Vesely (TURP)[9]	80	68.1 (mean), (±7.9)	TURP	24, 96	IPSS	IPSS-QoL	available	NA
Vesely (TUMT)[9]	102	67.9 (mean), (±8.4)	TUMT	24, 96	IPSS	IPSS-QoL	available	NA
Han[31]	71	68.2 (mean), 46–88	TURP	12–55, 19 (median)	IPSS	IPSS-QoL	available	available
Masumori[32]	34	NA	TURP	3, 36, 84, 144	IPSS	IPSS-QoL	NA	NA
Oh[33]	134	69.9 (mean), (±7.5)	TURP	12	IPSS	NA	available	available
Min[34]	285	69.3 (mean), (±8.2)	TURP	≥ 3	IPSS	IPSS-QoL	available	available

SD, standard deviation; QoL, quality of life; Q_max_, maximal flow rate on uroflowmetry; PVR, post-void residual; NA, not available; TURP, transurethral prostatectomy; TUMT, transurethral microwave thermotherapy; IPSS, International Prostate Symptom Score; PVP, photoselective vaporization of the prostate; BOO, bladder outlet obstruction; DUA, detrusor underactivity; ILC, interstitial laser coagulation.

### Comparison of BOO positive versus BOO negative patients

**Fig 2:**
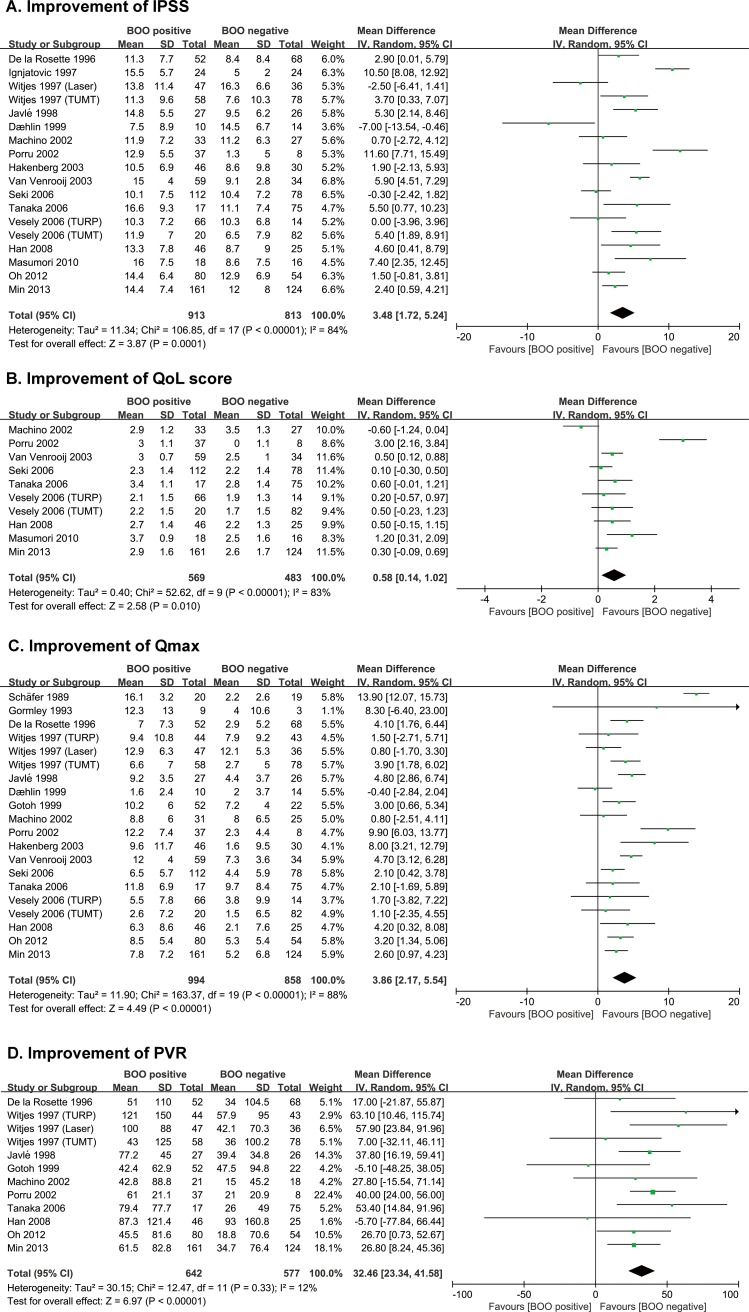
Forest plots comparing improvement of outcome parameters after transurethral surgery with or without bladder outlet obstruction (BOO) using random effects model. (A) Improvement of International Prostate Symptom Score (IPSS), (B) Improvement of quality of life score (QoL), (C) Improvement of maximal flow rate on uroflowmetry (Qmax), (D) Improvement of post-void residual volume (PVR).

Forest plots of the meta-analyses comparing the treatment outcome between urodynamic BOO positive and negative patients are shown in Fig 2. Each figure represents pooled MD and the respective 95% CI of ΔIPSS (Fig 2A), ΔIPSS-QoL (Fig 2B), ΔQ_max_ (Fig 2C), and ΔPVR (Fig 2D). In the comparisons of BOO positive and negative patients, all pooled MDs were significantly greater than zero: ΔIPSS (pooled MD, 3.48; 95% CI, 1.72–5.24; p < 0.01; studies, 16; participants, 1726; Fig 2A), ΔIPSS-QoL (pooled MD, 0.56; 95% CI, 0.14–1.02; p = 0.01; studies, 9; participants, 1052; Fig 2B), ΔQ_max_ (pooled MD, 3.86; 95% CI, 2.17–5.54; p < 0.01; studies, 17; participants, 1852; Fig 2C), and ΔPVR (pooled MD, 32.46; 95% CI, 23.34–41.58; p < 0.01; studies, 10; participants, 1219; Fig 2D). This means that BOO positive patients have better surgical outcomes in all parameters compared to BOO negative patients.

### Subgroup analysis

Subsequently, the subgroup analyses using patients who underwent TURP were performed (Table 3). In patients with TURP, the MDs were also statistically significant for all outcome parameters including ΔIPSS (pooled MD, 4.30; 95% CI, 2.25–6.35; p < 0.01), ΔIPSS-QoL (pooled MD, 0.59; 95% CI, 0.11–1.07; p = 0.02), ΔQ_max_ (pooled MD, 4.57; 95% CI, 2.47–6.67; p < 0.01), and ΔPVR (pooled MD, 33.30; 95% CI, 24.38–42.23; p < 0.01). Subgroup analyses of BOO comparisons by the two dominant criteria for BOO diagnosis (BOOI > 40 cmH_2_O, and lin PURR grade ≥ 2, 3, or 4) were also performed (Table 4). Except for the ΔIPSS-QoL (pooled MD, 0.21; p = 0.33; 95% CI, -0.21–0.64) of the BOOI subgroup, all pooled MDs of the outcome parameters were significantly greater than zero.

**Table 3:** Subgroup analysis in patients who underwent conventional transurethral resection of the prostate (TURP).

	No. of included articles	Included studies	No. of participants	Pooled MD (95% CI)	p value	I^2^	х^2^ (p value)
BOO positive vs. BOO negative							
Improvement of IPSS	13	[9,25–36]	1261	4.30 (2.25–6.35)	< 0.01	86%	84.05 (< 0.01)
Improvement of IPSS-QoL	9	[9,25,26,28–31,32,34]	950	0.59 (0.11–1.07)	0.02	85%	52.58 (< 0.01)
Improvement of Q_max_	15	[8,9,18,19,24–31,33,34,36]	1387	4.57 (2.47–6.67)	< 0.01	90%	137.14 (< 0.01)
Improvement of PVR	9	[8,24–26,30,31,33,34,36]	880	33.30 (24.38–42.23)	< 0.01	1%	8.06 (0. 43)

MD, mean difference; CI, confidence interval; BOO, bladder outlet obstruction; IPSS, International Prostate Symptom Score; QoL, quality of life; Q_max_, maximal flow rate on uroflowmetry; PVR, post-void residual.

**Table 4:** Sensitivity analysis of bladder outlet obstruction (BOO) comparisons using the two dominant criteria for BOO diagnosis (BOO index [BOOI] > 40 cmH2O, and linear passive urethral resistance relation [lin PURR] grade ≥ 2, 3, or 4).

	No. of included articles	Included studies	No. of participants	Pooled MD (95% CI)	p value	I^2^	х^2^ (p value)
Diagnosis by BOOI							
Improvement of IPSS	7	[25,27,28,31,33,34,36]	772	3.29 (1.51–5.06)	< 0.01	70%	19.89 (< 0.01)
Improvement of IPSS-QoL	4	[25,28,31,34]	509	0.21 (-0.21–0.64)	0.33	67%	9.02 (0.03)
Improvement of Q_max_	7	[25,27,28,31,33,34,36]	768	3.78 (2.60–4.95)	< 0.01	45%	10.87 (0.09)
Improvement of PVR	5	[25,31,33,34,36]	582	29.24 (17.50–40.98)	< 0.01	0%	1.61 (0.81)
Diagnosis by Lin PURR							
Improvement of IPSS	8	[8,20,23,26,29,30,32,35]	772	3.73 (0.05–7.40)	0.05	90%	82.50 (< 0.01)
Improvement of IPSS-QoL	4	[26,29,30,32]	361	1.19 (0.03–2.35)	0.04	92%	38.52 (< 0.01)
Improvement of Q_max_	7	[8,20,23,24,26,29,30]	851	2.84 (1.30–4.38)	< 0.01	68%	25.25 (< 0.01)
Improvement of PVR	5	[8,20,24,26,30]	637	34.28 (17.28–51.28)	< 0.01	41%	10.14 (0.12)

MD, mean difference; CI, confidence interval; BOOI, BOO index; IPSS, International Prostate Symptom Score; QoL, quality of life; Q_max_, maximal flow rate on uroflowmetry; PVR, post-void residual.

### Assessment of heterogeneity and publication bias

**Fig 3:**
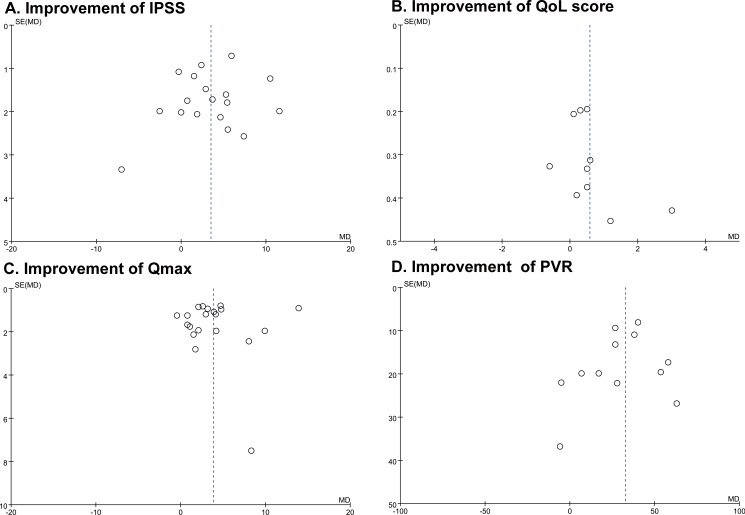
Funnel graphs of the assessment of potential publication bias in studies comparing improvement of outcome parameters after transurethral surgery with or without bladder outlet obstruction (BOO). (A) Improvement of International Prostate Symptom Score (IPSS), (B) Improvement of quality of life score (QoL), (C) Improvement of maximal flow rate on uroflowmetry (Qmax), (D) Improvement of post-void residual volume (PVR).

Despite our attempt to limit between-study heterogeneity through strict inclusion and exclusion criteria, heterogeneity between treatment outcomes still remained (heterogeneity х^2^ test: p < 0.05 in ΔIPSS, ΔIPSS-QoL, and ΔQ_max_ comparisons, I^2^ range: 12–88%; Fig 2). However, there was no clear evidence of funnel plot asymmetry for outcomes (Fig 3). Therefore, it can be concluded that there was no clear evidence of publication bias.

## Discussion

### Urodynamic BOO and surgical outcome

There have been insufficient evidences on which to base clear statements about ‘the right treatment’, despite a large number of studies published over several decades[40]. The preoperative evaluation of BPH patients provided clear examples of practice based on poor evidence. Recent international guidelines recommend surgical intervention and the decision to perform surgery primarily relies on the physician who should decide the best initial treatment on a case-by-case basis according to clinical conditions[1,2]. Poor correlation between the degree of urodynamic BOO and degree of patient symptoms is suggested by some researchers[41,42]. Those findings have indirectly supported the fact that UDS is not particularly useful as a preoperative evaluation in transurethral surgery for BPH.

However, if the basic principles of transurethral surgery are based on the classic BOO model, it can be expected that the degree of obstruction affects the treatment outcome. However, there has been not much evidence supporting this hypothesis[1]. The results of the current study provide a higher LE, which confirms the utility of performing UDS before transurethral surgery for BPH treatment.

During our survey of literature, we encountered various definitions of the urodynamic findings, especially for “BOO”. BOO is basically defined as methods of analyzing the pressure-flow plots. One of the major aims of pressure-flow study is to provide an objective diagnosis on weather the urethral resistance to flow is abnormally elevated[43]. For that purpose, methods have been developed to quantify pressure-flow plots in terms of one or more numerical parameters[13,14,44–49]. However, the optimal method for BOO diagnosis has not been confirmed[43]. Nevertheless, it seems that there is some degree of reliability on inter-test agreement due to the underlying similarity for diagnosing BOO[50]. Moreover, in our current study, subgroup analyses using the two dominant definition criteria demonstrated consistency except for the ΔIPSS-QoL of BOOI subgroup (Table 4).

### Limitations and strengths of the current study

Our study presents some limitations. First, none of the studies included in the current meta-analysis specified a prospective design. To the best of our knowledge, there has been no randomized study regarding the usefulness of UDS for guiding clinical management in male LUTS[1]. This may be due to the complexity of designing prospective study. It is difficult to draw any definitive conclusions when studies are not conducted prospectively. Thus, the results of the present study are important because, they can provide a higher LE regarding the diagnostic value of preoperative UDS in male LUTS patients who are being considered for transurethral surgery. The results of our study also can be an important reference for designing of further prospective studies in the future.

Second, due to the unavailability of mean and variance (or SD) data in some studies, those values are estimated using other presented distributional parameters for the outcome synthesis (see S2 Table). This can lead to some errors used in the estimation processes. However, the data imputation techniques used in this study were shown to have a low possibility of statistical errors in a previous study[51]. For the clarification of these points, accumulation of more evidence is needed.

Third, in our current meta-analysis, there was some heterogeneity in the included studies (Fig 2; especially in ΔIPSS, ΔIPSS-QoL, and ΔQ_max_ comparisons). Heterogeneity can be caused by numerous factors such as inclusion criteria, type of surgery, sample size, period of postoperative outcome evaluation, urodynamic cut-off values, and adjustment for other co-factors. It is also very difficult to explain inter-study heterogeneity, due to the variability in clinical characteristics across patients within studies. To lessen the heterogeneity related bias, we adopted the random-effect model for data synthesis, which is known to be to draw more conservative results[12]. Moreover, the direct evidence due to publication bias was not shown (Fig 3).

Lastly, the BOO negative group also can experience symptom improvements from BPH surgery, although the degree of improvement in the BOO negative group is significantly less than that in the BOO positive group (Fig 2). Therefore, urodynamic BOO (BOOI > 40 or lin PURR grade ≥ 2, 3, or 4 in this study) might not be an absolute indication for surgical treatment in patients with PBH. This indicates that urodynamic BOO positive patients with BPH who are considering surgery would have better treatment outcomes than BOO negative patients. However, being BOO positive (or negative) is not an absolute indication that the patient should (or should not) receive the surgery.

Despite some limitations, the findings from the present study suggest that preoperative urodynamic BOO has a diagnostic role in predicting treatment outcomes of surgery in male LUTS patients. The strengths of the current study are as follows: (1) broad, unbiased search of the literature; (2) strict criteria for study selection; and (3) application of standardized methods for systematic review[6,7]. Moreover, due to the relatively large number of eligible studies, subgroup (sensitivity) analysis could be performed according to the type of surgery (TURP) and the definition of BOO. Our subgroup analysis demonstrated consistency with the main results (Tables 3 and 4).

## Conclusions

In conclusion, our meta-analysis results demonstrated significant association between preoperative BOO positive patients and better improvement of surgical outcome parameters including IPSS, IPSS-QoL, Q_max_, and PVR. On these grounds, preoperative urodynamic BOO may have a diagnostic role in predicting treatment outcomes after surgery in male LUTS patients. However, the diagnostic value of UDS for preoperative evaluation also needs to be confirmed in prospective controlled trials before any definitive conclusions can be made.

## Supporting information

S1 Checklist The PRISMA checklist of this study (page 1). (TIF)

S2 Checklist The PRISMA checklist of this study (page 2). (TIF)

S1 Table Detailed query settings for search strategy. (DOCX)

S2 Table Related matters regarding processing the outcome parameters for data synthesis. (DOCX)
