# Supplementary figures and images for: Diagnostic value of urodynamic bladder outlet obstruction to select patients for transurethral surgery of the prostate: Systematic review and meta-analysis
Source: PLoS One. 2017 Feb 27;12(2):e0172590. doi: 10.1371/journal.pone.0172590 (PMC5328266; doi:10.1371/journal.pone.0172590)

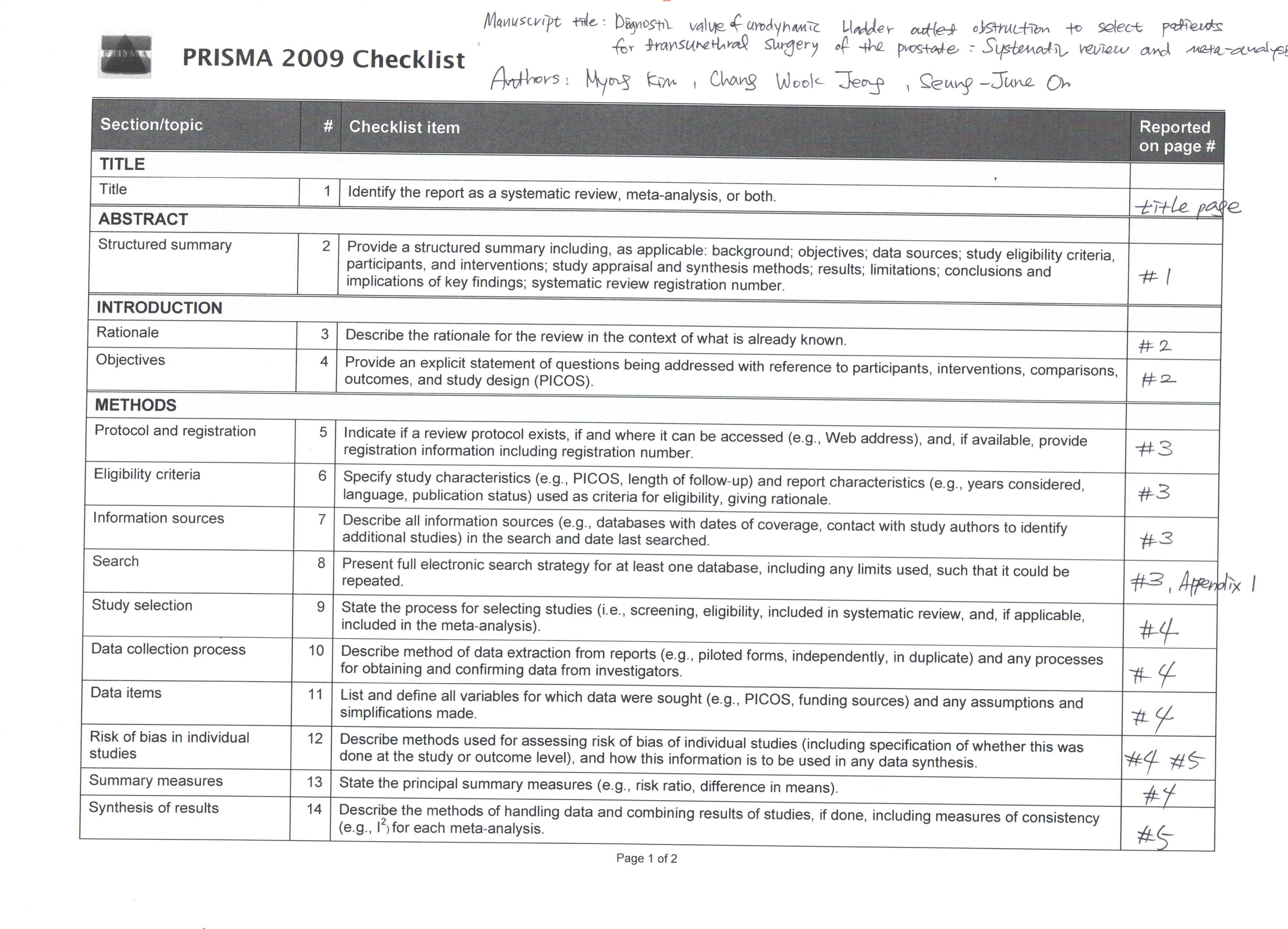

Supplement: S1 Checklist — (TIF) [file pone.0172590.s001.tif]

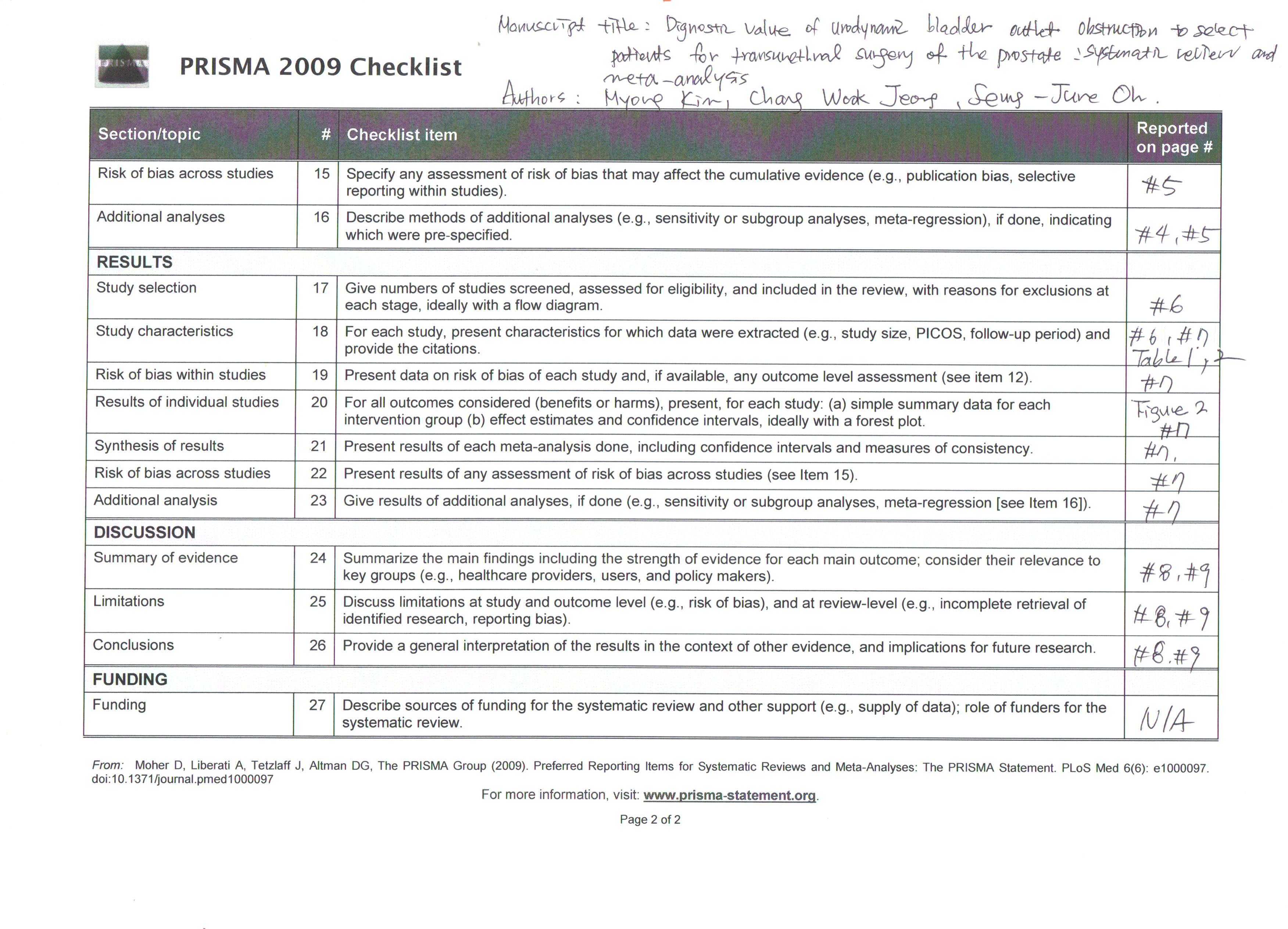

Supplement: S2 Checklist — (TIF) [file pone.0172590.s002.tif]
