# Supplementary material for: Diagnostic value of urodynamic bladder outlet obstruction to select patients for transurethral surgery of the prostate: Systematic review and meta-analysis
Source: PLoS One. 2017 Feb 27;12(2):e0172590. doi: 10.1371/journal.pone.0172590 (PMC5328266; doi:10.1371/journal.pone.0172590)
Supplement: S1 Table — (DOCX) [file pone.0172590.s003.docx]

S1 Table. Detailed query settings for search strategy

| Search | Query |
| --- | --- |
| #1 | Search "Prostatic Hyperplasia"[Mesh] |
| #2 | Search benign prostat* |
| #3 | Search lower urinary tract symptom |
| #4 | Search LUTS |
| #5 | Search (#3 or #4) |
| #6 | Search male |
| #7 | Search female |
| #8 | Search (#6 not #7) |
| #9 | Search man |
| #10 | Search woman |
| #11 | Search (#9 not #10) |
| #12 | Search (#8 or #11) |
| #13 | Search (#5 and #12) |
| #14 | Search (#1 or #2 or #13) |
| #15 | Search "Transurethral Resection of Prostate"[Mesh] |
| #16 | Search transurethral resection* |
| #17 | Search transurethral* |
| #18 | Search TURP |
| #19 | Search TUI* |
| #20 | Search (#15 or #16 or #17 or #18 or #19) |
| #21 | Search "laser" |
| #22 | Search holmium |
| #23 | Search thallium |
| #24 | Search potassium* titanyl* phosphate |
| #25 | Search KTP |
| #26 | Search Nd* YAG |
| #27 | Search PVP |
| #28 | Search vaporization |
| #29 | Search enucleation |
| #30 | Search HoLEP |
| #31 | Search ablation |
| #32 | Search (#21 or #22 or #23 or #24 or #25 or #26 or #27 or #28 or #29 or #30 or #31) |
| #33 | Search (#20 or #32) |
| #34 | Search (#14 and #33) |
| #35 | Search "Urodynamics"[Mesh] |
| #36 | Search urodynamic* |
| #37 | Search pressure* flow* |
| #38 | Search cystometry |
| #39 | Search obstruction |
| #40 | Search underactiv* |
| #41 | Search acontractile* |
| #42 | Search impaired detrusor* |
| #43 | Search (#35 or #36 or #37 or #38 or #39 or #40 or #41 or #42) |
| #44 | Search (#34 and #44) |

Presented as query form of Pubmed

Core logics of search queries were not different in other database searching.
