# Supplementary material for: Diagnostic value of urodynamic bladder outlet obstruction to select patients for transurethral surgery of the prostate: Systematic review and meta-analysis
Source: PLoS One. 2017 Feb 27;12(2):e0172590. doi: 10.1371/journal.pone.0172590 (PMC5328266; doi:10.1371/journal.pone.0172590)
Supplement: S2 Table — (DOCX) [file pone.0172590.s004.docx]

**S2 Table.** Related matters regarding processing the outcome parameters for data synthesis

| Study | Related matters | Data processing | Notes |
| --- | --- | --- | --- |
| Schäfer[18] | Sub-grouped in three, using the standards of P_muo_ < 25cm H_2_O, 25 – 40cm H_2_O, and > 40cmH_2_O | Outcome parameters of two groups (P_muo_ < 25cm H_2_O and 25 – 40cm H_2_O) were integrated using pooled mean, and SD[11] | - |
| Gormley[19] | Presented pre- and post-TURP Q_max_ of whole subject | Calculated mean ΔQ_max_ and SD using presented parameters[12] | - |
| De la Rosette[20] | Sub-grouped in three, using the standards of Lin PURR grade 0 – 1, 2 – 3, and 4 – 6 | Outcome parameters of two groups (Lin PURR grade 0 – 1, and 2 – 3) were integrated using pooled mean, and SD | - |
| Ignjatovic[35] | ΔIPSS was presented as median value with IQR | Mean and SD were estimated using presented median and IQR[11] | - |
| Witjes[8] | Symptom score of TURP subgroup was presented using Frimodt-Møller score  ΔIPSS was presented as median value with range  Each sub-group was categorized in three, by the standards of Lin PURR grade 0 – 1, 2 – 3, and 4 – 6 | Excluded from the data synthesis  Mean and SD were estimated using presented median and range[11]  Outcome parameters of two groups (Lin PURR grade 0 – 1, and 2 – 3) were integrated using pooled mean, and SD | Outcomes of each modality (TURP/PVP/TUMT) were integrated separately. |
| Javlé[36] | Outcome parameters were presented as mean value with SE  Sub-grouped in three, by the standards of BOOI < 20cm H_2_O (unobstructed), 20 – 40 cm H_2_O (equivocal), and > 40cmH_2_O (obstructed) | SD were estimated using presented SE and sample size[11]  Outcome parameters of two groups (unobstructed and equivocal) were integrated using pooled mean, and SD | - |
| Dӕhlin[23] | Outcome parameters were presented as median value with 95% CI | Mean and SD were estimated using presented sample size and 95% CI[11] | - |
| Gotoh[24] | Sub-grouped in three as group A (obstructed and normal detrusor), group B (obstructed and weak detrusor), group C (unobstructed and weak detrusor) | Outcome parameters of group A and B were integrated | - |
| Machino[25] | Presented pre- and post-TURP IPSS, IPSS-QoL,Q_max,_ and PVR with SD of each group | Estimated the mean Δ IPSS, Δ IPSS-QoL, ΔQ_max,_ and ΔPVR with their SD using pre- and post-TURP values | - |
| Porru[26] | Presented pre- and post-TURP IPSS, IPSS-QoL,Q_max,_ and PVR with SD of each group | Estimated the mean Δ IPSS, Δ IPSS-QoL, ΔQ_max,_ and ΔPVR with their SD using pre- and post-TURP values | - |
| Hakenberg[27] | Outcome parameters were presented as mean value with SE  Sub-grouped in three, by the standards of BOOI < 20cm H_2_O (unobstructed), 20 – 40cm H_2_O (equivocal), and > 40cmH_2_O (obstructed) | SD were estimated using presented SE and sample size  Outcome parameters of two groups (unobstructed and equivocal) were integrated using pooled mean, and SD | - |
| Van Venrooij[28] | ΔIPSS, ΔIPSS-QoL, and ΔQ_max_ were presented as median value with IQR | Mean and SD were estimated using presented median and IQR | - |
| Seki[29] | Presented pre- and post-TURP IPSS, IPSS-QoL, and Q_max,_with SD of each group  Presented 3 and 12 months postoperative data | Estimated the mean Δ IPSS, Δ IPSS-QoL, and ΔQ_max_ with their SD using pre- and post-TURP values  Utilized the 3 month postoperative data due to larger population | - |
| Tanaka[30] | Presented pre- and post-PVP IPSS, IPSS-QoL, Q_max,_ and PVR with SD of each group  Sub-grouped in three, by the standards of Lin PURR grade 0 – 1, 2 – 3, and 4 – 6 | Estimated the mean Δ IPSS, Δ IPSS-QoL, ΔQ_max,_ and ΔPVR with their SD using pre- and post-TURP values  Outcome parameters of two groups (Lin PURR grade 0 – 1, and 2 – 3) were integrated using pooled mean, and SD | - |
| Vesely[9] | Presented pre- and post-treatment IPSS, IPSS-QoL,and Q_max_ with SD of each group  Sub-grouped in three, by the standards of DAMPF < 43 (minor), 43 – 65 (moderate), and > 40cmH_2_O (severe)  Presented 3 and 12 months postoperative data | Estimated the mean Δ IPSS, Δ IPSS-QoL, and ΔQ_max_ with their SD using pre- and post-treatment values  Outcome parameters of two groups (minor and moderate) were integrated using pooled mean, and SD  Utilized the 3 month postoperative data due to larger population | Outcomes of each modality (TURP/ TUMT) were integrated separately. |
| Han[31] | Presented pre- and post-TURP IPSS, IPSS-QoL, and Q_max,_with SD of each group | Estimated the mean Δ IPSS, Δ IPSS-QoL, and ΔQ_max_ with their SD using pre- and post-TURP values | - |
| Masumori[32] | Presented pre- and post-TURP IPSS, and IPSS-QoL with SD of each group  Presented 3, 36, 72, and 144 months postoperative data | Estimated the mean Δ IPSS and Δ IPSS-QoL with their SD using pre- and post-TURP values  Utilized the 3 month postoperative data due to larger population | - |
| Oh[33] | IPSS-storage and IPSS-emptying were presented separately  Presented pre- and post-TURP IPSS, Q_max,_ and PVR with SD of each group | IPSS-total was calculated using pooled mean, and SD  Estimated the mean Δ IPSS, ΔQ_max_, and ΔPVR with their SD using pre- and post-TURP values | - |
| Min[34] | Sub-grouped in three, by the standards of BOOI < 20cm H_2_O (unobstructed), 20 – 4 0cm H_2_O (equivocal), and > 40cmH_2_O (obstructed) | Outcome parameters of two groups (unobstructed and equivocal) were integrated using pooled mean, and SD | - |

P_muo,_ minimal urethral opening pressure; SD, standard deviation; TURP, transurethral prostatectomy; Q_max_, maximal flow rate on uroflowmetry; Lin PURR, linear passive urethral resistance relation; IPSS, International Prostate Symptom Score; IQR, interquartile range; PVP, photoselective vaporization of the prostate; TUMT, transurethral microwave thermotherapy; SE, standard error; BOOI, bladder outlet obstruction index; CI, confidence interval; QoL, quality of life; PVR, post-void residual; DUA, detrusor underactivity; DAMPF, Detrusor Mean Lin PURR Factor; IPSS-storage, sum of IPSS question 2, 4, and 7; IPSS-emptying, sum of IPSS question 1, 3, 5, and 6; IPSS-total, sum of total IPSS question
